# Editorial: Marine biomolecules

**DOI:** 10.3389/fchem.2015.00052

**Published:** 2015-08-19

**Authors:** Antonio Trincone, Mikhail Kusaykin, Svetlana Ermakova

**Affiliations:** ^1^Istituto di Chimica Biomolecolare, Consiglio Nazionale delle RicerchePozzuoli, Italy; ^2^G.B. Elyakov Pacific Institute of Bioorganic Chemistry, Far Eastern Branch of the Russian Academy of SciencesVladivostok, Russia

**Keywords:** marine biomoleculers, marine oligosaccharides, marine compounds, marine enzymes, marine biocatalysts

Even before the development of the idea to exploit marine biomolecules for human needs (pharmaceuticals, cosmetics, etc.), the interest for such compounds is implicit when one ponders the astonishingly basic definition of marine biotechnology as “a natural extension of cultural practices of garnering food from the ocean” by the anthropologist Helmreich who beautifully summarizes the origins of marine biotechnology and its deep significance to human society (Helmreich, 2003). Free from any sectarian, discipline-directed bias for a particular type of molecules and, as a consequence of this inclusive unfiltered consideration of all types of molecule, is the great flexibility of the biological adaptation of marine organisms to the wide range of environmental conditions found (temperature, salinity, tides, pressure, radiation, light, etc.). Indeed this adaptation has empowered marine livings with an enormous reservoir of any type of precious biological material for both basic research and biotechnological improvements. Relative to the terrestrial well-known ecosystem, the prevalent and unknown marine environment is valued as a source of enzymes exhibiting new functions and activities to fulfill human needs (Trincone, 2013), as well as other biomolecules such as important polysaccharides which are the most abundant renewable biomaterial found in oceans. This list cannot be completed without the inclusion of small molecular weight compounds characterized by various molecular skeletons isolated from marine organisms (sponges, corals, and other marine invertebrates), which possess interesting activities. Moreover, it is still widely accepted that the assignment of precise biological functions to genes, proteins, and enzymes in marine environment is the least developed aspect.

The above consideration was the motivation for the proposal of this Research topic that aims to centralize review contributions, idea and comments simply related to all marine biomolecules. In particular, results of enzymatic bioprospecting in a gross marine environment were expected, along with research for structural characterization and biological function of marine polysaccharides and all kind of research related to the complexity of bioprocesses in marine environments, with an inter- and multi-disciplinary approach. Probably the short lifetime of the research topic hampered a more crowded collection of different contributions spanning all class of biomolecules. Nevertheless, 78 authors from the most prestigious research institutions from different parts of world have submitted articles reporting original results along with reviews, opinions, and perspective contributions all composing this e-book. Two new research topics dedicated to marine oligosaccharides and to marine compounds in food domain, are directly derived from this success and currently active in Frontiers in Marine Science under the Specialty of Marine Biotechnology.

In order to understand the mechanism of action of the α-galactosidase of the marine bacterium *Pseudoalteromonas* sp. KMM 701, the stereochemistry of its hydrolytic reaction has been analyzed by ^1^H NMR spectroscopy for the identification of the first anomer of the sugar formed, before mutarotation equilibrium. The data showed that the enzymatic hydrolysis of substrates proceeds with retention of configuration due to a double displacement mechanism of reaction. Hydrolysis of terminal α-linked galactose residues from glycoconjugates has found a number of useful potential biotechnological and medical applications with direct research involvement by the same group of authors (Balabanova et al., 2010). Worldwide efforts focused on the subject are also recently reviewed (Bakunina et al., 2014).

From the same prestigious Russian institution, G.B. Elyakov Pacific Institute of Bioorganic Chemistry, two other groups have contributed to this Research topic with a review and an opinion article. The review presented a survey in collaboration with the Department of Biochemistry, Dong-A University, South Korea, on marine triterpene glycosides having very low toxicity and considered potential suitable agents for the prevention and treatment of cancer. The conclusion affirms that fine details of the structural characteristics including carbohydrate moiety and sulfation are involved in the biological activities of marine triterpene glycosides and the study is essential for developing marine drugs.

The opinion article evaluates the status of different research on marine polysaccharides. The molecular diversity of these molecules is very interesting with new structures and, except those used traditionally in food and non-food industries, with functionalities that are unknown and unexplored so that biomolecular tools for study in this field are very important. The authors specially focused on fucoidan and fucoidanases on which the Russian group is intensively working (Kusaykin et al., 2008; Vishchuk et al., 2011, 2013; Silchenko et al., 2013; Shevchenko et al., 2015). Despite the obvious prospects for exploitation in medicine, fucoidan has not yet been declared a drug and the question of the title makes sense in view of the biological effects of a food supplement called Fucolam® recently registered in Russia that in addition to the immunomodulatory, antibacterial, antiviral, and antitumoral activities, has probiotic, hepatoprotective, glucose, and cholesterol lowering effects.

The importance of the carbohydrate class of biomolecules in marine environment is highlighted by the presence of other contributions. An original article from the Station Biologique de Roscoff, France, reports on an iota-carrageenan sulfatase detected in the cell-free lysate of the marine bacterium *Pseudoalteromonas carrageenovora*. Not only do the rheological properties of these carrageenans depend on their sulfate content but the enzymatic manipulation of sulfates is an enabling technology of paramount importance for modulation of activity of sulfate containing biomolecules.

Furthermore, from the Laboratory of Marine Biotechnology and Microbiology Division of Applied Marine Life Science of Hokkaido University, Japan, a study is presented on an excellent source of carbohydrate active marine enzymes, the *Aplysia* genus. In this case the characterization of a cellulase, from the common sea hare *Aplysia kurodai* is the topic related to the importance of marine carbohydrate biomasses as a source material for ethanol fermentation.

A review directly linked to this topic is provided by the National Institute of Renewable Energy, Punjab, India, detailing recent findings and advanced developments of algal biomass for biofuel production, highlighting the great impact of fossil fuels on carbon cycle (carbon balance) related to combustion. According to this green chemistry approach that aims to bypass competition with agricultural food and feed production, researchers, and entrepreneurs have focused interest on algal biomass and other marine bioresources as alternative feed-stock for improved production of biofuels.

Turning back to small molecular agents, the very interesting perspective article from the Institute of Biomolecular Chemistry, Italy, is worth mentioning last. Some of the volatile terpenoids found in marine sponges and nudibranchs have also been found in terrestrial plants and insects. Being odorant molecules that are hydrophobic in nature, they cannot be effective in any form of remote sensing based on diffusion in water. Olfaction is generally regarded as a distance sense, while gustation is a contact sense, and exactly the same volatile molecules, that are almost insoluble in water, would be considered at the same time as being odorant on land, and tasted by contact at sea. After an in-depth analysis from ecological, chemical, and biological perspective of the actual view about selection of odorant receptors for hydrophobic interactions, the authors report a new perspective aiming at a radical solution preserving the usual taste-smell dichotomy.

## Conflict of interest statement

The authors declare that the research was conducted in the absence of any commercial or financial relationships that could be construed as a potential conflict of interest.
